# Vessel Geometry Estimation for Patients with Peripheral Artery Disease

**DOI:** 10.3390/s24196441

**Published:** 2024-10-04

**Authors:** Hassan Saeed, Andrzej Skalski

**Affiliations:** 1 Department of Measurement and Electronics, AGH University of Krakow, 30-059 Krakow, Poland; hsaeed@agh.edu.pl; 2 MedApp S.A., 30-037 Krakow, Poland

**Keywords:** centerline, geometry measurements, peripheral arteries, distance transform, 3D imaging

## Abstract

The estimation of vessels’ centerlines is a critical step in assessing the geometry of the vessel, the topological representation of the vessel tree, and vascular network visualization. In this research, we present a novel method for obtaining geometric parameters from peripheral arteries in 3D medical binary volumes. Our approach focuses on centerline extraction, which yields smooth and robust results. The procedure starts with a segmented 3D binary volume, from which a distance map is generated using the Euclidean distance transform. Subsequently, a skeleton is extracted, and seed points and endpoints are identified. A search methodology is used to derive the best path on the skeletonized 3D binary array while tracking from the goal points to the seed point. We use the distance transform to calculate the distance between voxels and the nearest vessel surface, while also addressing bifurcations when vessels divide into multiple branches. The proposed method was evaluated on 22 real cases and 10 synthetically generated vessels. We compared our method to different state-of-the-art approaches and demonstrated its better performance. The proposed method achieved an average error of 1.382 mm with real patient data and 0.571 mm with synthetic data, both of which are lower than the errors obtained by other state-of-the-art methodologies. This extraction of the centerline facilitates the estimation of multiple geometric parameters of vessels, including radius, curvature, and length.

## 1. Introduction

Peripheral artery disease (PAD), which causes a significant decrease in blood flow to the legs and causes structural and functional abnormalities in muscle tissue, is the third most prevalent atherosclerotic cardiovascular disease after coronary artery disease and stroke [1]. It affects around 10% of adults over the age of forty in America [2].

There is an increased incidence of cardiovascular events among patients with PAD [3]. Cardiovascular disease contributed the most to deaths in 2019, constituting 32% of total deaths in that year, and increased by 42% from baseline in 1990 [4]. Epidemiological studies have reported that more than 200 million patients suffer from PAD worldwide; a large proportion was contributed by the elderly population [5]. In addition, PAD is relatively common among more than 65-year-old individuals with the age-related variable of obesity and diabetes [6]. Therefore, a timely quantitative diagnosis and assessment of PAD risk can be beneficial in prolonging human life expectancy and improving quality of life.

To address the issues posed by PAD, the use of stenting has reduced the risk of elastic recoil, restenosis, and flow-limiting dissections associated with conventional balloon angioplasty [7]. However, stenting has its own set of problems, including in-stent restenosis ISR [8], and the possibility of early and late stent thrombosis [9].

Due to these constraints, drug-coated balloons (DCBs) were developed as a unique therapy method for PAD. DCBs operate by rapidly delivering antiproliferative medicines to the vessel wall through the inflation of a single balloon, keeping to the notion of ‘leaving nothing behind’ [10,11]. The concept behind DCB technology is to treat coronary lesions using balloons and medications to reduce restenosis rates [12,13]. DCBs have evolved as a novel technique for percutaneous coronary intervention (PCI), demonstrating promising results for in-stent restenosis (ISR) and small vessel disease [14,15]. According to Ullah et al. [16], using DCBs to treat symptomatic lower extremity PAD results in reduced restenosis rates, improved patency, and a reduced need for repeat revascularization. DCBs also result in fewer adverse events and limb amputations, while not increasing the risk of ‘all-cause mortality’ [16].

Given the significant advances in DCB technology, an accurate geometric assessment of peripheral arteries remains crucial. Centerlines of blood vessels are useful tools for making important anatomical measurements, such as length and diameter [17]. Key elements such as the integration of vessel centerlines, the planning of the surgical path, and the analysis of geometric parameters are essential tools in vascular treatments. In particular, focusing on geometric parameters such as vessel length and radii is important in treatment planning  [18]. Even the most skilled surgeons face difficulties in determining the appropriate surgical path due to the unpredictable nature of the anatomical features and delicate operative circumstances. Accurate vascular radius estimations help avoid problems such as inadvertent tissue damage during catheter insertions. This precision increases the localization of the problem area and, as a result, the surgical outcomes. Despite the importance of these geometrical parameters, there is a substantial lack of research on their extraction for PAD. This study presents an innovative approach for the geometric analysis of arteries affected by PAD, validated using real and synthetic datasets, which can be used to assist surgeons in both the preoperative and intraoperative stages.

## 2. Related Work

### 2.1. Machine Learning/Deep Learning-Based Approach

Recent advances have seen the application of deep learning methods, with many proposed in recent years. Convolutional neural networks (CNNs) have been shown to be adept at capturing complicated properties in vascular architecture [19]. Dorobanțiu [20] used the 3D U-Net architecture to segment the coronary artery centerline with voxel-level accuracy by classifying each voxel as belonging to the centerline. However, because the output may not always be a single voxel wide, a thinning technique is used as a post-processing step to ensure a one-voxel-thick centerline. This thinning phase is a deterministic method used to refine the output of the deep learning model. Similarly, Rjiba et al. [21] used a binary classification network-driven local vessel filter to effectively extract centerlines by analyzing the position of the centerpoint of the current patch relative to the centerline. However, there are still several challenges. Firstly, the approach involves breaking down full Computed Tomography Angiography images into smaller patches for training, which introduces a significant amount of non-vascular information into the process. As a result, this procedure is laborious and time consuming. Furthermore, like other methods, it faces issues such as vessel discontinuities, which require post-processing techniques such as connected component analysis to preserve the completeness of the centerline. Additionally, the coordinates of the centerline points predicted by the network can be imprecise, as there are often differences between the centerpoint of the patch and the actual centerline point. To overcome this, the pipeline uses deterministic methods in both pre-processing (vessel enhancement) and post-processing, ensuring a connected, anatomically consistent centerline tree.

In paper [22], the authors provide a comprehensive pipeline for rib segmentation, labeling, and anatomical centerline extraction from CT data. The procedure begins with segmentation based on deep learning, which involves segmenting and labeling the ribs individually. The centerlines are then retrieved using skeletonization techniques such as TEASAR and L1-medial. In their study [23], Wolterink proposed a CNN-based orientation classifier designed to assess the direction of the arteries and then extract the centerlines. Their methodology involves mapping various points on a sphere to different directions, and the direction classification process assists identify probable points corresponding to each direction. However, in the post-processing phase a pruning step is applied to correct potential leakage into adjacent non-coronary vessels, where long centerlines are shortened and pruned based on the vessel’s smallest estimated radius. This integration of post-processing ensures an anatomically correct centerline. Mostafa et al. [24] developed an automated workflow utilizing a regression U-Net to automatically recognize coronary ostia and employed the enhanced CNN Tracker to track and extract the endpoint. Notably, their method introduced a weight term in the loss function allowing them to calculate the angle between the predicted vector and each sample vector, which considerably improved the direction classifier’s performance. Furthermore, Yang et al. [25] described a discriminative coronary artery tracking method that alternates between training a tracker and a discriminator. Their discriminator plays a crucial role in the establishment of a robust learning-based stopping criterion that efficiently distinguishes coronary arteries from other cardiac tissues. However, it should be noted that the classification techniques utilized in these studies have a rather low accuracy. To achieve higher accuracy, the proposed models would need to increase the number of sampling points, which unfortunately results in significant memory consumption for training parameters. Additionally, the outcomes relating the extraction of multiple branches and distal parts are not as adequate as anticipated, which means they need further post-processing. Moreover, a large amount of vessel annotation findings need to be provided, which requires a long time and a great deal of vitality in manual annotation.

### 2.2. Deterministic-Based Approach

Deterministic-based methods are particularly beneficial when data are scarce since they do not require large databases for training, as opposed to deep learning methods, which rely substantially on extensive labeled data to achieve high accuracy. Insufficient training data may significantly impact the performance of deep learning models, leading to overfitting or poor generalization. In contrast, deterministic methods rely on well-defined algorithms and mathematical principles, allowing them to work consistently even with little or no training data. This makes deterministic methods practical and efficient in circumstances where obtaining a large number of high-quality labeled data can be challenging or impracticable.

State-of-the-art vessel centerline generation techniques utilizing Voronoi diagrams have shown considerable promise. For coronary artery tracking, study [26] employed an automated 3D Voronoi-based centerline extraction approach. In their study, the researchers divided a 3D image into numerous 2D slices, applied the Voronoi algorithm to each slice, and then stacked the resulting 2D centerlines to create 3D medial surfaces. The authors of [18] also utilized Voronoi diagrams for centerline extraction, using the Vascular Modeling Toolkit (VMTK) to obtain the centerlines. VMTK, an open source library used to study vascular structure and extract centerlines from medical imaging data, uses an approach based on Voronoi diagrams [27]. It is integrated as an extension with 3D Slicer [27], an open source platform for medical image informatics and visualization, thus expanding its capabilities. VMTK retrieves centerlines by creating a Voronoi diagram within the vessel volume and determining the medial axis using distances to the vessel surface. Study [28] proposed a fully automated technique to extract vascular tortuosity characteristics in the supraaortic area, which is critical for planning stroke treatment. Centerlines are retrieved from a binary map by finding the shortest pathways between extremal locations and then using a Voronoi diagram to improve accuracy by minimizing a wave propagation integral. This technique seeks to increase the efficiency and efficacy of vascular analysis in clinical settings. However, objects with irregular boundaries can generate dense Voronoi diagrams that frequently need substantial pruning of the medial surface to reach a precise centerline, making the process computationally intensive.

Another state-of-the-art approach [29] employs skeletonization to extract the centerlines based on Lee [30], while in [31], the authors used skeletonization to determine the endpoints of the vessels. The study in [32] aimed to automate the identification of vessel segments in the Circle of Willis using pathfinding algorithms. Centerlines were retrieved by first segmenting the vessels using a 3D seeded-region growing method, then skeletonizing the segmented binary masks with the scikit-image Python module to obtain the centerlines. However, the skeleton technique is not optimal for extracting centerlines, since it generates multiple false centerlines in complicated structures, resulting in inaccuracies and artifacts. Liu et al. [33] introduced a completely automated approach for minimum path tracking that integrates direction features from atlas-based models with inertia features from previously identified centerline points. Other methods, such as a Bayesian tracking algorithm that uses adaptive particle filters [34], have also been explored. However, the particle filtering approach experiences difficulties due to high computational demands, resulting in extended processing times, leaving room for further adjustments in accuracy. In our previous research [35], we proposed a method for extracting centerlines from coronary datasets within a segmented 3D medical binary volume. The process begins by generating a distance map using the Euclidean distance transform, which is then smoothed using a Gaussian filter. The Gaussian scale, adjusted for each dataset, is crucial for accurate smoothing and feature enhancement. Then, a Hessian matrix is computed for each voxel based on this map. The Hessian matrix eigenvalues and eigenvectors are analyzed to identify tubular structures, with the eigenvector corresponding to the smallest eigenvalue indicating the direction of the centerline of the vessel.

Existing centerline extraction methods, such as Voronoi-based algorithms, often generate erroneous centerlines in the presence of irregular vessel borders, necessitating significant post-processing. These methods may result in centerlines that deviate from the vessel structure, particularly when faced with complex or noisy data, or completely miss smaller branches, reducing the reliability of the extracted geometry. Similarly, skeletonization techniques can produce incorrect centerlines, resulting in spurious or false branches. In the same way, image intensity-based methods using Hessian matrix computations rely on the correct Gaussian scale for smooth centerline extraction; incorrect scaling risks missing fine features or magnifying noise. Such errors may have a significant impact on the planning and implementation of treatment.

Furthermore, deep learning-based algorithms are significantly based on large annotated datasets for training, potentially limiting their use to other imaging modalities. These methods may also fail to generalize well when applied to unseen datasets or new types of imaging. In contrast, the method proposed in this article is deterministic, which means that it does not depend on any training dataset, which makes it very flexible to a variety of imaging modalities and not bound to the peripheral arteries. This characteristic allows for a more consistent and accurate extraction of geometric parameters without the biases introduced by machine learning models trained on specific datasets.

## 3. Method

### 3.1. Proposed Solution

To estimate the geometry of a vessel, an approach to extract the centerline is proposed that takes a segmented 3D volume as input. The procedure begins with the use of a distance transform algorithm to build a distance map from the binary mask, which calculates the shortest distance between each voxel and the nearest object boundary, allowing for the quick identification of the object’s medial axis. Next, the binary mask’s skeleton is extracted, which gives us an approximate estimation of the vessel’s medial axis, often, however, with false branches. The user then identifies the seed point and goal points from the list of centerpoints generated by the algorithm, enabling interactive data extraction and the determination of the centerline in regions of interest. The algorithm then runs the breadth-first search algorithm (BFS) on the skeleton to create a depth map. This depth map is used to traverse from each goal point toward the seed point, removing extraneous branches and determining the optimum path. The algorithm then utilizes this optimal path to track and traverse from the seed location to each goal point, while utilizing the maximum distance transform value. The proposed approach is presented in Algorithm 1.
**Algorithm 1:** Smoothened Centerlines From Seed Point Toward Goal Points
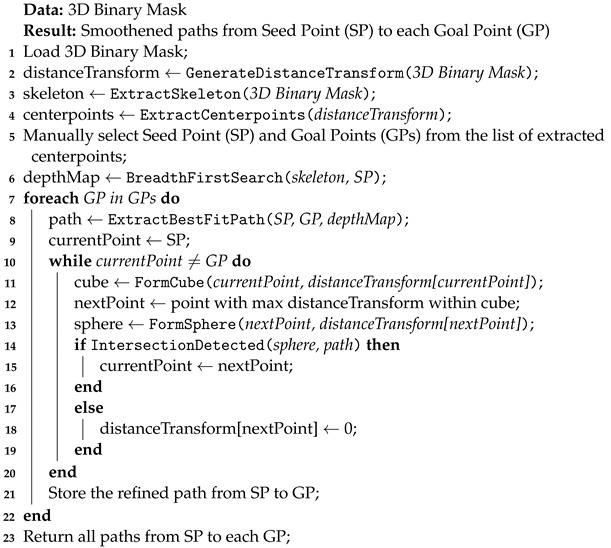


#### 3.1.1. Euclidean Distance Transform

The distance transform is utilized to extract the geometric characteristics of the vessel since it computes the shortest distance between each voxel and the closest object border. This enables the exact characterization and measurement of the vessel’s geometry, including diameter, length, and branching patterns. These geometrical parameters are critical for understanding the spatial geometry of the vascular system, locating the pathology, and selecting the optimal access point to the vessel. Following the process in [35,36], given a 3D binary mask of shape a×b×n, if a voxel (p,q,r) is in the foreground, that is, if a pheripheral artery is found, set it to 1; otherwise, set it to 0. The distance transform computes the distance between all voxels equal to one and their nearest voxels equal to zero, according to (1) and (2):

(1) $$dt_{p,q,r}=\underset{\left(x,y,z\right)\in B_{r}}{min}DT[(p,q,r),(x,y,z)]$$

(2) $$DT[(p,q,r),(x,y,z)]=\sqrt{{\left(p-x\right)}^{2}+{\left(q-y\right)}^{2}+{\left(r-z\right)}^{2}}$$

where (p,q,r) and (x,y,z) are the voxel coordinates of the input binary data [35]. The searching sphere is indicated by $B_{r}$, which has a radius of *r* and an initial value of *r* set to 1. If r = 1, a sphere with a diameter of 2 and a center at voxel (p,q,r) are generated. The voxels on the ball’s surface are examined. If a voxel is identified with value 0, the search is paused to compute the distance using (2). The distances between all targeted voxels in the peripheral artery are calculated using the 3D Euclidean distance transform. The 3D binary array will then be translated into a distance map where the target–voxel value is larger than 0 and the distance is greater than 1 [35]. Consequently, the local maximum values are located exactly in the center of the vessels.

#### 3.1.2. Skeletonization

**Figure 1:**
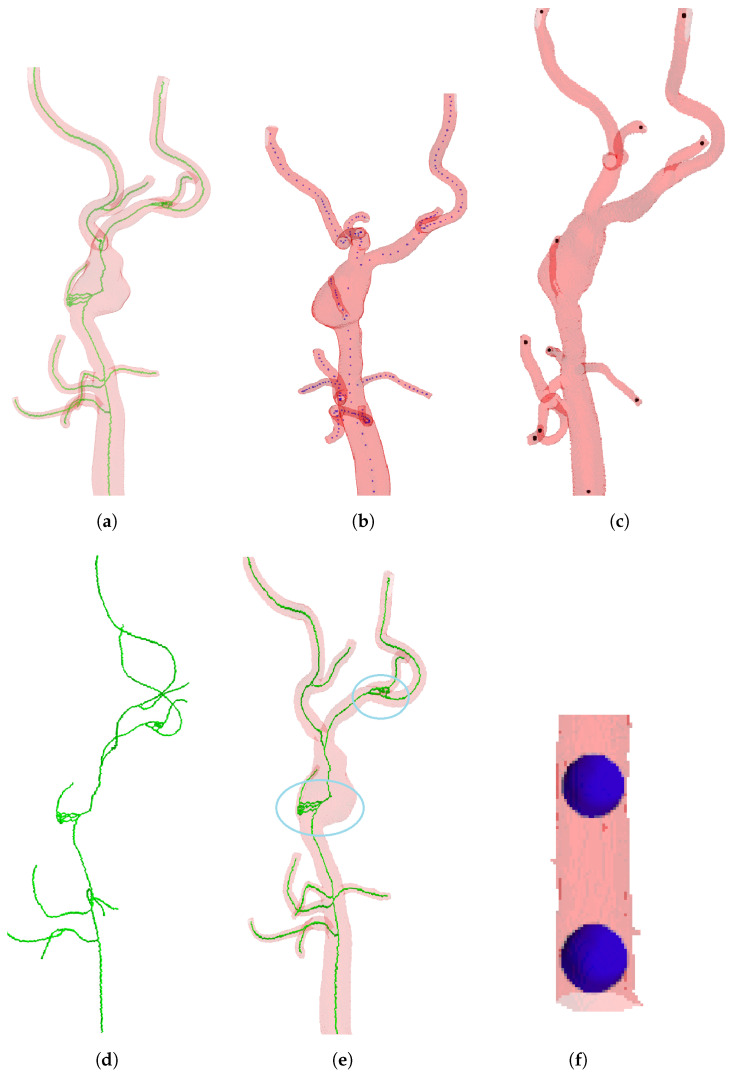
Peripheral artery structure and analysis. (**a**) Three-dimensional binary mask and extracted skeleton. (**b**) Centerpoints extracted in binary mask. (**c**) User-extracted seed and goal points. (**d**) Traversed skeleton via BFS alogrithm. (**e**) Spurious branches from skeletonization. (**f**) Spheres formed around the centerpoint.

In the following phase of the algorithm, skeletonization is used to extract the medial axis of the binary mask, providing a preliminary estimate. Following the approach in [32], the skeleton is extracted from the 3D binary mask. A 3D binary mask of the peripheral artery with its extracted skeleton is shown in Figure 1a.

#### 3.1.3. CenterPoint Extraction

In the subsequent step of the method, centerpoints are generated within the binary mask using the distance transform to correctly determine the medial axis. In the distance transform array, the highest value and the corresponding index (i,j,k) are identified. This highest value indicates the largest distance from the background, indicating a potential centerpoint [35]. As illustrated in Figure 1f, the sphere is created according to (3), with a radius (*r*) equal to the value of the maximum distance transform and its center at the corresponding index (i,j,k).

(3) $${\left(i-x\right)}^{2}+{\left(j-y\right)}^{2}+{\left(k-z\right)}^{2}\leq r^{2}$$

where (x,y,z) refer to all the voxels that satisfy (3).

The list stores the coordinate (i,j,k) of the maximum distance transform values. Following this, the maximum distance transform value at (i,j,k) is set to 0, and any additional voxels in the distance transform array that satisfy Equation (3) at the current centerpoint are set to 0 [35]. This process is carried out until the maximum value of the distance transform array reaches zero. We now have a complete list of all the centerpoints. All extracted centerpoints determined in the peripheral artery are shown in Figure 1b.

#### 3.1.4. Seedpoint and Goalpoint Selection

In the next stage of the algorithm, determining the injection (seed) and target (goal) points is critical for procedural planning, especially for catheter insertion. The seed point acts as a starting point for tracing paths to specific goal points, which are facilitated by user interaction with 3D data to identify points of interest. Both the seed and goal points, which are derived from previously defined centerpoints, coincide with the vessel’s central axis, ensuring exact localization and eliminating the possibility of selecting points that do not correspond to the vessel’s medial axis. The seed point and goal point chosen by the user can be seen in Figure 1c.

#### 3.1.5. Three-Dimensional Skeleton Traversal

Next, the BFS method aims to meticulously investigate and traverse the 3D space, starting from a seed point and a goal within a three-dimensional skeleton structure. It carefully examines nearby nodes at the current depth level before progressing to the next, using a queue to track nodes in the order they are discovered. As it traverses the structure, BFS marks each node to prevent revisiting and generates potential moves based on predetermined parameters such as boundaries and obstacles. This process continues until the algorithm reaches the goal node or exhausts all viable pathways. Figure 1d illustrates a skeleton traversed by the implemented BFS algorithm.

Skeletonization algorithms are susceptible to challenges, including the generation of false branches within an acquired skeleton. This significant issue is caused by the presence of noise or inconsistencies in the data that are being skeletonized, which results in the formation of irrelevant branches that incorrectly represent the actual structure. In addition, artifacts inherent in medical imaging exacerbate the situation by contributing errors that appear as extraneous branches in the skeletonized representation. Figure 1e shows exemplary false branches generated through skeletonization.

#### 3.1.6. Optimizing Traversal: Best Path to Overcome False Branches on BFS Skeleton Maps

When traversing a map generated by a breadth-first search (BFS) of a skeletonized structure, it is common to encounter numerous false branches. To address this issue, employing a “best path” methodology from the destination (goal) back to the start point can be beneficial.

The “best path” approach involves constructing a path within the skeletal structure that accurately mirrors the intended trajectory while filtering out irrelevant branches, aiming for the most accurate representation possible.

Using a BFS-generated depth map, the algorithm traces a path from the goal point. At each step, it advances to a centerpoint with a depth value that is one less than the current centerpoint depth value. The trace ends when the seed point with depth = 0 is found, at which point the best path is constructed counting the steps backward from the goal to the seed point.

**Figure 2:**
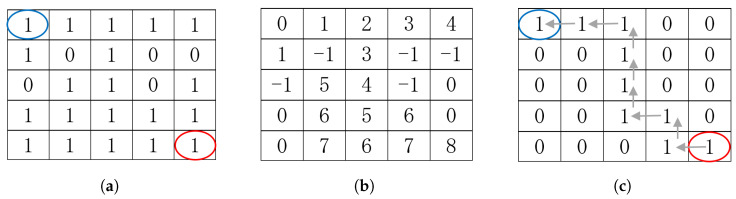
Employing BFS on a binary mask, generating a depth map, and then extracting an optimal path from the goal (encircled in red) to the seed point (encircled in blue). (**a**) Binary mask. (**b**) Depth map. (**c**) Path from goal to seed.

For an enhanced comprehension of fundamental concepts, a simplified 2D example is presented in Figure 2. The binary mask is depicted, with 1 denoting the foreground (object) and 0 representing the background shown in Figure 2a. The starting point, indicated at [0, 0] and encircled in blue, is referred to as the seed point, while the goal point at [4, 4] is encircled in red within a 5 × 5 matrix. Subsequently, Figure 2b displays the corresponding depth map obtained after applying BFS to the binary mask. It can be noticed that the background in the depth map is represented with a value of −1 for clarity and to avoid confusion. In this generated depth map, the seed point has a depth of 0, while the goal point at the coordinates [4, 4] has a depth of 8. Figure 2c illustrates the navigation using the best path from the goal point to the seed point, where the path is represented by arrows. This path is extracted from the depth map generated in Figure 2b.

**Figure 3:**
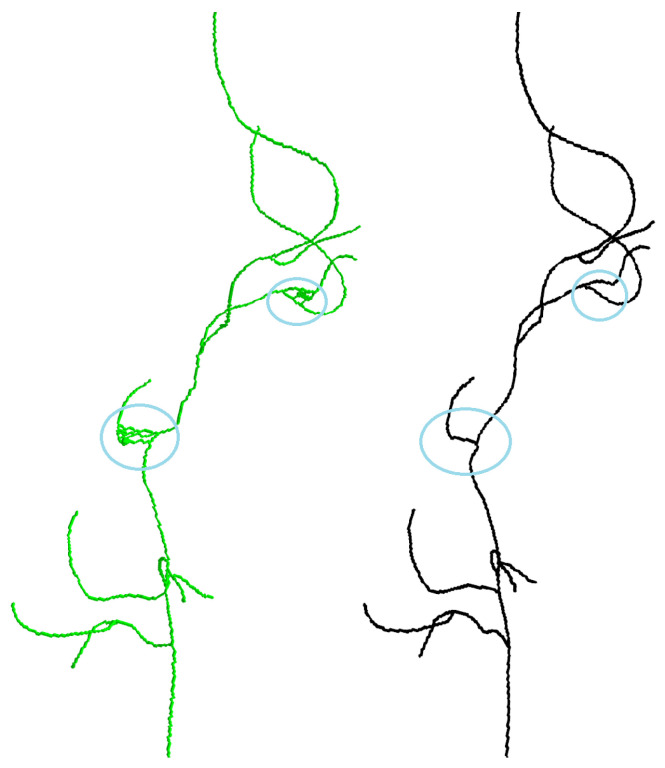
Side-by-side comparison of skeletonization (**left**) and best-path result on skeleton (**right**).

Figure 3 shows a portion of the peripheral artery with two representations: skeletonization and the best path. It is evident that using the best-path method helps eliminate false branches.

#### 3.1.7. Optimizing Path Extraction Algorithm for Geometric Parameters from Seed to Goal

**Figure 4:**
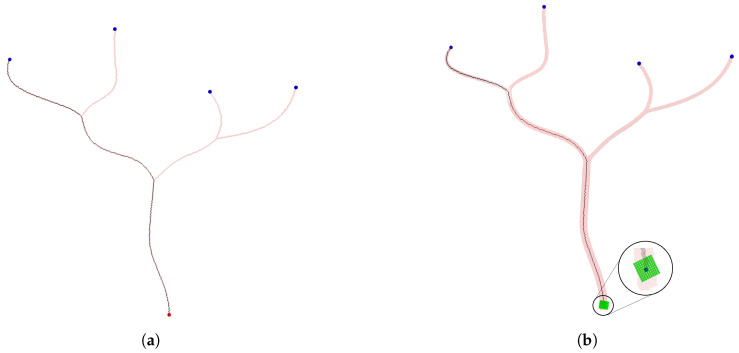
Vessel length measurement and six sub-arrays forming cube around centerpoint visualization. (**a**) Best path from seed to goal shown on the skeleton. (**b**) Cube centered around the seed point, illustrating the spatial region of interest.

Once the paths have been optimized, the algorithm will traverse from the seed to the goal point as illustrated in Figure 4a while tracking the maximum distance values from the distance map. The best path guides the direction of this traversal. This directed route is then used to obtain geometric parameters from this generated centerline. Starting at the seed point, it creates a cube with a radius equal to the distance transform value (r) at the current position with coordinates (x, y, z) and the 3D binary mask size ($size_{x},size_{y},size_{z}$) represented in Figure 4b. The range of the cube is calculated according to (4) and the cube’s bounds are check with (5). The cube, with its simple geometric shape and well-defined edges and corners, is suitable for this purpose. The edges of the cube also correspond to the cardinal directions, making it easier to determine the centerline direction of the vessel [35]. Consequently, a cube is utilized to track the maximum distance transform from the seed point to the goal point.

The cube range is calculated as follows:

(4) $$a_{i}=\left\{\begin{array}{cc} a-r & \mathrm{if}\;i=1\\ a+r & \mathrm{if}\;i=2 \end{array}\right.\;\mathrm{for}\;a\in \left\{x,y,z\right\}$$

The range boundary is checked as follows:

(5) $$\begin{array}{ccc} \mathrm{if}\;a_{1}<0: & a_{1} & =0\\ \mathrm{if}\;a_{2}\geq {\mathrm{size}}_{a}: & a_{2} & ={\mathrm{size}}_{a}-1 \end{array}\;\mathrm{for}\;a\in \left\{x,y,z\right\}$$

**Figure 5:**
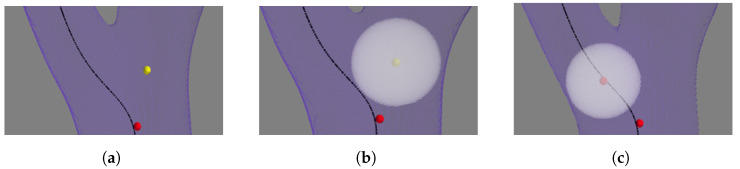
Algorithm update step to address bifurcation and follow best path. (**a**) Max distance point detected off best path. (**b**) Algorithm checking for best-path intersection. (**c**) Algorithm following updated direction.

After forming the cube as described in Equations (4) and (5), which is represented by six arrays (one for each face of the cube), the algorithm fills each array with the distance transform values from the corresponding coordinates on the 3D distance map. For each array, the algorithm identifies the coordinates of the next potential centerpoint with the maximum distance transform value and selects the point with the highest distance value, represented by the yellow point in Figure 5a. The algorithm then creates a sphere with a radius equal to the distance value at this yellow point, as shown in Figure 5b, and checks for intersections with the best path, which is used as the reference direction to move from the seed toward the goal. If no intersection is detected, indicating a bifurcation site, the algorithm discards this maximum distance point, returns to the bifurcation (the previous centerpoint) indicated by the red point in Figure 5b, sets the distance value of the discarded point (in yellow) to 0, and selects another point with the next highest distance transform value. This process repeats, especially in the case of multiple bifurcations, until an intersection is found with the best path, ensuring that the algorithm advances in the correct direction, as shown in Figure 5c.

The algorithm continues its progression until the detection of the goal point. This technique is iterated for each goal point. As a result, the 3D route path includes all pathways from the seed toward each of the goal points.

### 3.2. Bifurcation Handling

Many vessel tracking algorithms struggle with bifurcation, which refers to the branching of vessels. To address this issue, the same approach is applied as in our previous research in [35]. The proposed algorithm generates a centerlineMap initialized to zeros with the same shape as the 3D binary mask. When tracking the centerline from the seed point to a specific goal point, the coordinates of the centerline are retrieved, and the corresponding positions in the centerlineMap are set to 1 [35]. In subsequent iterations, the centerline within the centerlineMap grows from the new goal point to the seed. The growth process halts if a value of 1, indicating an existing centerline, is encountered in the centerlineMap. If no such value is found, the corresponding indices in the centerlineMap are updated to 1. By avoiding overlap with previously created centerlines, the proposed algorithm’s bifurcation detection ensures a one-voxel-thick centerline.

### 3.3. Vessel Length Measurements

In the next phase, our algorithm determines the length of the vessels. Figure 4a shows the seed and goal points, where the red point represents the seed point, and the goal points are represented in blue. The length of the extracted centerlines is calculated using the depth map to determine the best path from the seed to the goal point, as can be seen in Figure 4a, where black points represent all the points between the seed and the selected goal, followed by an estimate of the Euclidean distance between subsequent points along this path.

## 4. Results and Discussion

### 4.1. Data Description

The entire algorithm was implemented in Python (version 3.9.7). Our platform was a common PC with 11th Gen Intel® Core™ i7, 2.3 GHz (Intel, Santa Clara, CA, USA). We tested the performance of our approach on data accessible in the Vascular Model Repository (VMR) [37,38], an open source database that contains cardiovascular cases. We specifically chose data on the architecture of the abdominal aorta as beyond this point in the provided database, the artery divides into two distinct iliac arteries, each serving one leg. This is significant in the context of peripheral artery health, as the iliac arteries connect to the peripheral arteries by branching into the external iliac artery, which becomes the femoral artery, the primary source of blood to the legs and lower body. This structural complexity is particularly relevant for evaluating centerline extraction algorithms, which must accurately trace vascular paths even through bifurcations. In total, we analyzed 22 cases that span a wide variety of ages, as summarized in Table 1.

We validated our algorithm’s results against the manually annotated centerpoints provided by VMR for each dataset and compared the performance of our algorithm with other state-of-the-art methods. By comparing with state-of-the-art methods, we extracted centerlines using the Voronoi-based approach through VMTK as described in [18] and the skeletonization method from the Python scikit-learn library (version 0.18.3) as outlined in [32].

To assess performance, the Euclidean distances between the centerline points extracted by each method and the manually annotated reference points (ground truth) were calculated. The average of these distances was then calculated to offer a quantitative evaluation of the accuracy. This average distance is an important performance metric that indicates how well the extracted centerlines from the proposed and other methods correspond with the ground truth, allowing us to objectively evaluate and compare the accuracy and performance of the proposed algorithm to other approaches.

**Figure 6:**
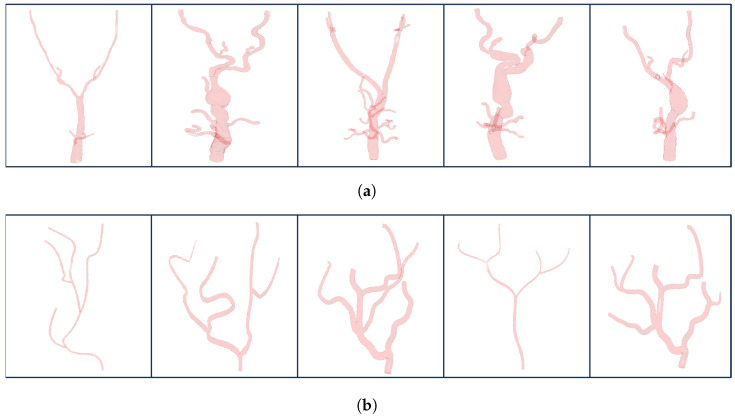
Examples of VMR dataset and synthetic data. (**a**) Exemplary cases of real patient data. (**b**) Exemplary cases of synthetic data.

Furthermore, synthetic data were also generated to further compare the performance of the proposed algorithm with other methods and manual annotations by evaluating the average error with respect to the ground truth. Figure 6 displays five example cases of both the VMR dataset and synthetic data.

### 4.2. Vascular Model Repository Data

According to the VMTK documentation [39], the centerline extraction module provides two options: network extraction and centerline tree extraction. Network extraction is a rapid and approximate way to extract a full centerline network, whereas centerline tree extraction is a precise Voronoi-based approach that uses an input surface and endpoints. For comparison, we retrieved centerlines with both options.

Table 2 provides a detailed comparison of the average error for the proposed algorithm and other methods for the extraction of the centerline on the VMR dataset. The errors are measured in millimeters (mm) and are benchmarked against the provided ground truth points.

**Figure 7:**
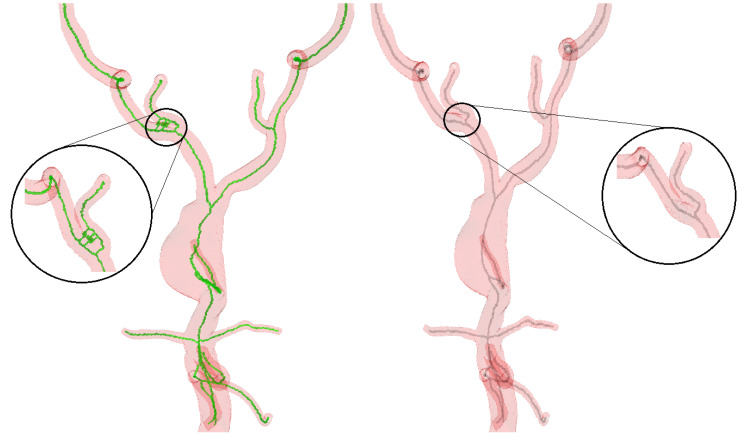
Side-by-side comparison of skeletonization (**left**) and proposed algorithm result (**right**).

Compared to other algorithms, the proposed method performs better, indicated by a lower average error, as shown in Table 2. The skeletonization method often generates erroneous branches that do not accurately represent the centerline of the vessel. For example, although dataset-12 in Table 2 shows a lower average error for the skeletonization method, it also contains erroneous branches that do not accurately represent the central path of the vessel, as illustrated in Figure 7.

**Figure 8:**
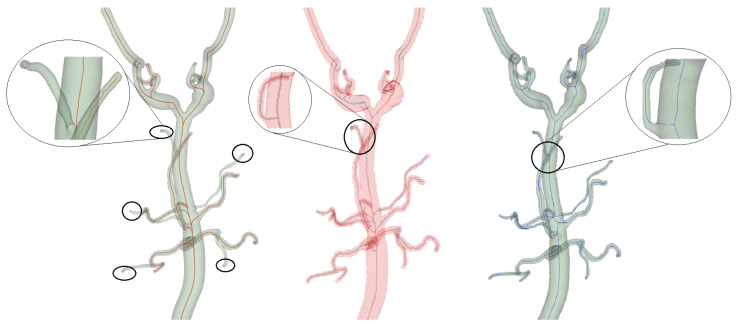
Side-by-side comparison of the VMTK centerline model **(left**)—the magnified view shows small vessel centerlines missed. Proposed algorithm (**center**)—capturing accurate and complete centerlines and the VMTK network model (**right**) with off-center centerlines that extend beyond the model’s boundary.

In contrast, centerline extraction using Voronoi-based methods through the slicer VMTK extension faced some challenges, such as incomplete extraction across some vessel branches despite manually adjusting the endpoints, and in some areas, the generated centerlines were not only off center but extended beyond the boundaries of the binary model, both of which are illustrated in Figure 8.

**Figure 9:**
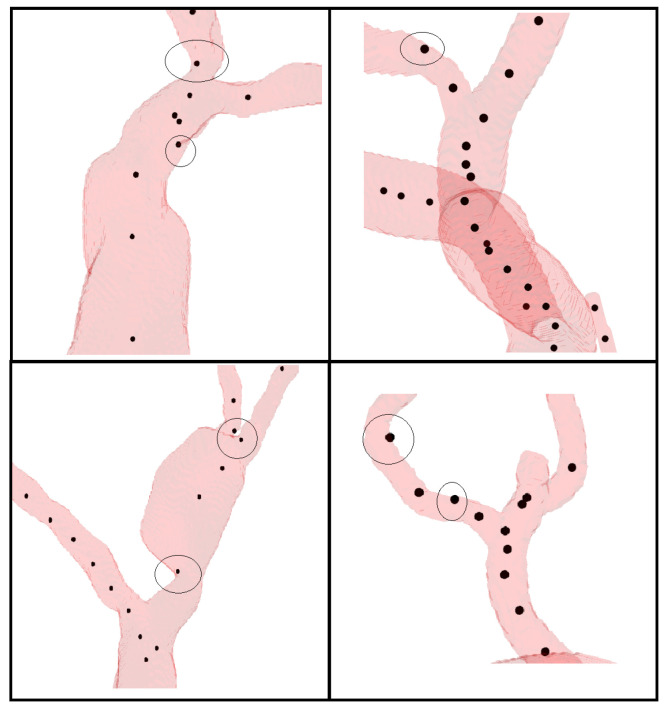
The image illustrates various data points from the VMR database with poorly annotated points that are misaligned and not centered on the vessel.

While working with the VMR dataset, inconsistencies were encountered in the manually annotated ground truth points provided with each dataset, as some of these points did not accurately represent the center of the vessels. This is illustrated in Figure 9. This variability influenced the evaluation of the centerline extraction methods, as demonstrated by the overall mean and standard deviation (STD) values observed in Table 2. Despite these constraints, our method exhibited a lower average error than other methods, indicating a better accuracy in centerline extraction. However, a higher STD across techniques, notably for VMTK-based outcomes, demonstrates how erroneous ground truth points can enhance variability in error measures. Addressing these annotation challenges is critical to enhancing the reliability and consistency of future assessments.

### 4.3. Synthetic Data

**Figure 10:**
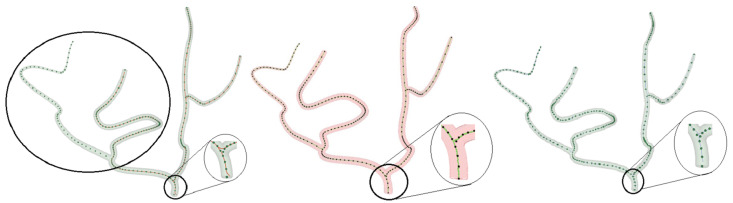
Side-by-side comparison of the VMTK centerline model (**left**)—the encircled area shows missed vessel centerlines, and the zoomed-in area reveals centerlines not passing through centerpoints at bifurcations; the proposed algorithm (**center**); and the VMTK network Model (**right**)—the zoomed-in part shows centerlines not passing through centerpoints at bifurcations—on synthetic data.

While working with the VMR dataset, we discovered discrepancies in the manually annotated ground truth points, as seen in Figure 9. In some cases, these annotations failed to correctly identify the center of the vessels. This underscores that depending on manual annotations, which are prone to human intravariability, intervariability, and mistakes, is not the ideal approach. To address this issue, we generated synthetic vessel data with bifurcations to represent peripheral arteries. This synthetic data include accurate ground truth, allowing us to compare the performance of the proposed centerline extraction algorithm with other methods. Figure 10 illustrates how the centerlines obtained by the proposed algorithm and other methods intersect with the ground truth. Furthermore, we manually annotated the centerlines with ImageJ, a popular image processing application specialized for scientific multidimensional images, to show the difference between ground truth and manual annotations. It is important to note that the discrepancy between the ground truth and the manually annotated points is greater than the errors produced by three of the four methods demonstrated in Table 3.

To generate the synthetic data, Bezier splines with the desired shape and number of bifurcations were first created using the open source 3D modeling application Blender. The spline form was altered using handles. The generated splines provided centerpoint data and dataset length information.

Table 3 presents data for ten datasets with the performance of each algorithm compared to the ground truth, with a summary of the average error and standard deviation at the end. Manual annotation is not only time consuming, but is also prone to errors attributed to human error, annotator variability, and the difficulty of reliably determining the precise centerpoint location in complicated structures.

The proposed method had a lower average error and a much smaller standard deviation (0.571 ± 0.025) than other methods, showing better accuracy and consistency in centerline extraction.

We compared the lengths extracted by the proposed algorithm from each synthetic dataset against the ground truth, with all measurements in millimeters (mm). The overall average error is summarized in Table 4.

Vessel centerline extraction improves blood vessel visibility in medical images, making it easier for healthcare practitioners to examine vessel anatomy and health. The centerline allows for the calculation of the plane and geometry of the vessel, which helps in the diagnosis and treatment of vascular diseases such as stenosis and aneurysms by providing precise measurements [35]. It is critical in interventional radiology and procedures such as angiography to guide catheters and medical equipment through the circulatory system, allowing less intrusive treatments [35].

**Figure 11:**
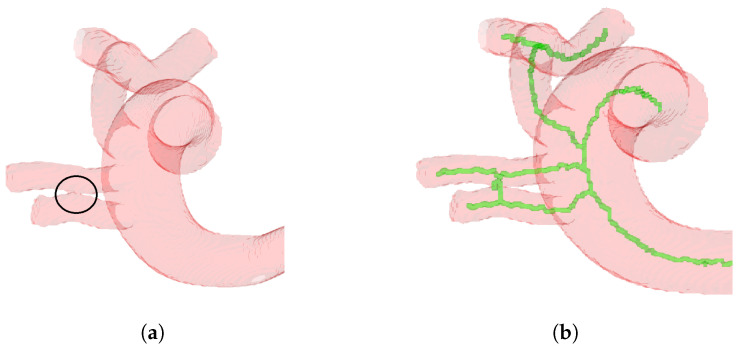
Dependence of centerline extraction on segmentation quality: Accurate segmentation yields reliable results; inaccurate segmentation yields unreliable results. (**a**) Poor segmentation causing incorrect vessel connections, with encircled vessels improperly joined. (**b**) False positive skeleton branch due to erroneous vessel connection, emphasizing the segmentation’s impact on accuracy.

The effectiveness of centerline extraction is inextricably tied to the results of segmentation. Accurate segmentation leads to robust and consistent centerline extraction, highlighting the importance of high-quality segmentation [35]. However, improper segmentation might also result in erroneous skeleton branches, reducing centerline accuracy and overall results, as can be seen in Figure 11. Assessing arterial disease, such as plaque or calcification, from a binary perspective can also be difficult, emphasizing the importance of accurate segmentation [35]. Distance transform-based centerline extraction approaches are adaptable to a variety of imaging modalities, including MRI, CT, ultrasound, and angiography. Unlike cutting-edge approaches, our methodology constantly creates centerpoints in the center using the distance transform, thereby decreasing erroneous centerline branches [35]. This adaptability enables precise vascular examination in a variety of therapeutic contexts. Furthermore, the centerline extraction approach described here is not restricted to blood vessels but can be used on a variety of structures in medical imaging.

In comparing the performance of our method on real versus synthetic datasets, several key differences emerged. Synthetic data, generated under controlled conditions, are often free of noise, irregularities, and artifacts. This control over the data environment simplifies annotation, as vessel dimensions and characteristics are predefined and known. The performance of each method on the synthetic data, including manual annotation, is shown in Table 3. Manual annotation is not an ideal option, as seen by the overall mean and standard deviation, which indicates higher variability and error. This emphasizes how manual annotation is heavily dependent on the annotator, making it a less reliable option.

For real datasets, we compared our method against the provided manual annotations, which, as demonstrated with the synthetic data manual annotation, is not the ideal approach. Real datasets present additional challenges, including noise, artifacts, and variability in vessel morphology. Manual annotation becomes even more difficult due to the complex and variable geometry of the peripheral arteries. As shown in Figure 9, some centerpoints were inaccurately annotated due to these complexities.

The proposed method has a direct effect on clinical treatment, particularly in the treatment of peripheral artery disease (PAD). Accurate centerline extraction is critical for planning stent placements, as precise vessel measurements ensure appropriate stent selection and lower complication hazards. During catheter-based interventions, reliable vessel paths guide catheter placement, curtailing the risk of vessel wall injury.

## 5. Conclusions

In this research, we present a novel method for centerline extraction based on the distance transform that achieves the reliable extraction of the centerlines in peripheral arteries. Our approach was validated on 22 real human cases from a VMR database and 10 synthetic cases, demonstrating better results with a lower average error than other state-of-the-art methods.

To address the challenges of manual annotation, we generated 10 synthetic vessel datasets incorporating complex bifurcations for the further validation of our approach. The manual annotation of synthetic data underscored its labor-intensive nature and susceptibility to human error and variability, as evidenced by significant average errors. In contrast, our method consistently produced an accurate centerline with significantly smaller average error and standard deviations. In addition, the effectiveness could also be proved in the comparison of the estimated vessel length measurements from the synthetic data against the provided ground truth.

The effectiveness of centerline extraction is closely related to the quality of segmentation. Accurate segmentation is essential for robust and consistent centerline extraction. As Figure 11 illustrates, inadequate segmentation can lead to inaccurate centerlines and lower accuracy. Therefore, improving the quality of the segmentation is crucial to enhancing the robustness and reliability of the proposed method.

Unlike deep learning-based methods, our deterministic approach does not require a training dataset and can be easily adapted to various anatomical structures, making it a versatile tool in medical imaging. However, it has been applied mainly to segmented data. Future research will focus on the extension of this method to nonsegmented data to broaden its clinical applicability.

In summary, our approach advances the field of the geometric analysis of arteries toward a reliable and adaptable solution that may have the potential to significantly improve the outcomes of vascular intervention, particularly in the treatment of peripheral artery disease.

## Figures and Tables

**Table 1:** Analysis of instances by gender and age.

	Male	Female	Total
Number of instances	18	4	22
Average age (years)	63.64 ± 14.40		

**Table 2:** Performance comparison in millimeter of vessel centerline extraction algorithms on vascular model repository (VMR) dataset.

Dataset	Proposed Algorithm	Skeletonization	VMTK Centerline Model	VMTK Network Model
1	0.967	1.100	16.049	1.496
2	1.425	1.441	2.174	2.345
3	1.216	1.307	1.969	2.132
4	1.741	1.858	3.688	2.669
5	1.613	1.654	6.271	2.030
6	1.476	1.451	1.694	2.314
7	1.673	1.674	1.964	2.492
8	1.015	1.204	2.316	2.844
9	1.223	1.266	2.070	2.126
10	1.222	1.311	2.922	1.686
11	1.383	1.448	1.552	2.288
12	2.076	1.744	4.844	3.426
13	1.341	1.338	2.893	2.213
14	1.011	1.028	2.833	1.802
15	1.548	1.600	4.489	1.844
16	1.008	1.008	36.805	1.560
17	1.884	1.931	5.357	2.968
18	1.358	1.477	2.044	2.280
19	1.066	1.170	1.082	1.778
20	1.125	1.221	8.670	1.649
21	1.651	1.757	2.357	2.349
22	1.375	1.453	4.191	1.713
Mean ± STD	1.382 ± 0.304	1.429 ± 0.263	5.374 ± 7.740	2.182 ± 0.492

**Table 3:** Performance comparison in millimeters of vessel centerline extraction algorithms on synthetic data.

Dataset	Proposed Algorithm	Skeletonization	VMTK Centerline Model	VMTK Network Model	Manual
1	0.547	0.566	9.30	2.690	0.757
2	0.538	0.593	111.427	0.368	1.105
3	0.587	0.808	1.30	2.495	2.013
4	0.604	0.628	32.818	1.027	2.118
5	0.577	0.772	0.768	1.959	1.886
6	0.573	0.623	23.520	1.100	1.457
7	0.599	0.773	0.816	2.484	1.964
8	0.547	0.607	4.866	0.752	1.050
9	0.546	0.582	16.544	0.372	1.110
10	0.595	0.839	0.739	1.258	1.242
Mean ± STD	0.571 ± 0.025	0.679 ± 0.106	20.210 ± 33.910	1.450 ± 0.889	1.470 ± 0.487

**Table 4:** Vessel length average error (mm).

Dataset	Average Error
1	0.61
2	2.59
3	0.84
4	1.64
5	0.96
6	1.20
7	0.84
8	0.98
9	2.74
10	0.77

## Data Availability

The data used in this study were accessed from the open Vascular Model Repository (VMR) available at https://www.vascularmodel.com/index.html (accessed on 25 June 2024). Access to synthetic data is available upon reasonable request.
